# Prognostic Factors of Survival of Advanced Liver Cancer Patients Treated With Palliative Radiotherapy: A Retrospective Study

**DOI:** 10.3389/fonc.2021.658152

**Published:** 2021-07-28

**Authors:** Qingling Hua, Dejun Zhang, Yunqiao Li, Yue Hu, Pian Liu, Guangqin Xiao, Tao Zhang, Jun Xue

**Affiliations:** ^1^Cancer Center, Union Hospital, Tongji Medical college, Huazhong University of Science and Technology, Wuhan, China; ^2^Department of Geriatrics, Union Hospital, Tongji Medical College, Huazhong University of Science and Technology, Wuhan, China

**Keywords:** liver cancer, palliative radiotherapy, prognostic factors, nomograms, multivariable Cox regression

## Abstract

**Aims:**

Survival benefit of liver cancer patients who undergo palliative radiotherapy varies from person to person. The present study aims to identify indicators of survival of advanced liver cancer patients receiving palliative radiotherapy.

**Patients and Methods:**

One hundred and fifty-nine patients treated with palliative radiotherapy for advanced liver cancer were retrospectively assessed. Of the 159 patients, 103 patients were included for prediction model construction in training phase, while other 56 patients were analyzed for external validation in validation phase. In model training phase, clinical characteristics of included patients were evaluated by Kaplan-Meier curves and log-rank test. Thereafter, multivariable Cox analysis was taken to further identify characteristics with potential for prediction. In validation phase, a separate dataset including 56 patients was used for external validation. Harrell’s C-index and calibration curve were used for model evaluation. Nomograms were plotted based on the model of multivariable Cox analysis.

**Results:**

Thirty-one characteristics of patients were investigated in model training phase. Based on the results of Kaplan-Meier plots and log-rank tests, 6 factors were considered statistically significant. On multivariable Cox regression analysis, bone metastasis (HR = 1.781, *P* = 0.026), portal vein tumor thrombus (HR = 2.078, *P* = 0.015), alpha-fetoprotein (HR = 2.098, *P* = 0.007), and radiation dose (HR = 0.535, *P* = 0.023) show significant potential to predict the survival of advanced liver cancer patients treated with palliative radiotherapy. Moreover, nomograms predicting median overall survival, 1- and 2-year survival probability were plotted. The Harrell’s C-index of the predictive model is 0.709(95%CI, 0.649-0.769) and 0.735 (95%CI, 0.666-0.804) for training model and validation model respectively. Calibration curves of the 1- and 2-year overall survival of the predictive model indicate that the predicted probabilities of OS are very close to the actual observed outcomes both in training and validation phase.

**Conclusion:**

Bone metastasis, portal vein tumor thrombus, alpha-fetoprotein and radiation dose are independent prognostic factors for the survival of advanced liver cancer patients treated with palliative radiotherapy.

## Introduction

Liver cancer (LC) is one of the most common malignancies and the fourth leading cause of cancer-related death worldwide (1). When diagnosed with LC, about 70% patients are in advanced stage, resulting in a poor five year overall survival (OS) rate(approximately 5%) (2). Most advanced LC patients are incurable (3), thereafter it is important to provide beneficial non-curative therapies for those patients (4, 5).

Palliative radiotherapy is one of the non-curative treatments for LC, which mainly aims to alleviate symptoms and improve the quality of life (QoL) (3, 6). Interestingly, occasional success of survival prolongation can be seen in patients receiving palliative radiation. More intensive treatment regimens should be recommended if those patients who will benefit from palliative radiotherapy can be identified. Inversely, patients with poor prognosis should not receive too much radiation treatment (7, 8). Therefore, it is essential to identify predictive indicators for the survival of advanced LC patients treated with radiation, which will be convenient for clinicians to distinguish those patients who will benefit from palliative radiotherapy.

We performed a retrospective study to develop several predictive factors and construct a predictive model by analyzing the characteristics of LC patients receiving palliative radiation. Nomograms for the survival prediction of cancer patients have been widely used. It can help clinicians to predict the prognosis of cancer patients by using a convenient numerical estimation model instead of complex statistical models. In this study, nomograms were also plotted based on the identified predictive factors and model.

## Methods

### Patient Selection

We retrospectively evaluated LC patients treated with palliative radiotherapy in our hospital between January 2017 and July 2020 following inclusion criteria: 1) age ≥ 18 years; 2) diagnosed by pathology or clinical evidences; 3) palliative radiotherapy for tumor in liver or distant metastatic sites; 4) stage IIIB-IV according to Chinese stage system of LC. The exclusion criteria consist of 1) curative radiotherapy; 2) metastatic tumor in liver from other cancers. The most recent follow-up date was July 26, 2020. Included patients were divided to be analyzed in model training phase and external validation phase. The training set comprised 103 advanced LC patients receiving palliative radiotherapy between November 2017 and July 2020. The external validation set comprised 56 similar patients treated between January 2017 and July 2020.

Clinical characteristics such as age, sex, stage, metastasis, radiation techniques, radiation dose, toxicity and other 25 variables were collected. Toxicities were recorded in accordance with the CTCAE (version 5.0). The primary end point was OS that was calculated from the time of palliative radiation to the time of death, or last follow-up (July 26, 2020). The requirement for informed consent from the patients was waived since this study is retrospective.

### Statistical Analysis

Statistical analysis was performed by using R software packages(version 3.6). Median follow-up time was estimated by reverse Kaplan-Meier curves. Kaplan-Meier curves and log-rank test were used to identify the factors associated with OS, and statistically potential factors were confirmed by multivariable Cox regression analysis. Proportional hazards (PH) assumption of multivariable Cox regression analysis was checked by Kaplan-Meier plot for category variables, while schoenfeld residuals were calculated for continuous variables (9). To confirm the assumption of proportionality, time-dependent covariate analysis was used. Martingale residual was calculated to assess nonlinearity of continuous variables. The continuous variables will be converted to category variables if they have no linearity. Variables with high co-linearity were not included in the same regression model. Hazards ratio (HR) and 95% confidence interval (CI) were calculated for each of the variables. Then, predictive model for OS was developed by using multivariable Cox regression analysis. After that, nomograms were constructed based on the predictive model (10). Harrell’s C-index and calibration curves were used to assess the feasibility of the predictive model (11). A two-tailed P value of <0.05 was considered statistically significant.

## Results

### Clinical Characteristics of Included Patients

**Figure 1:**
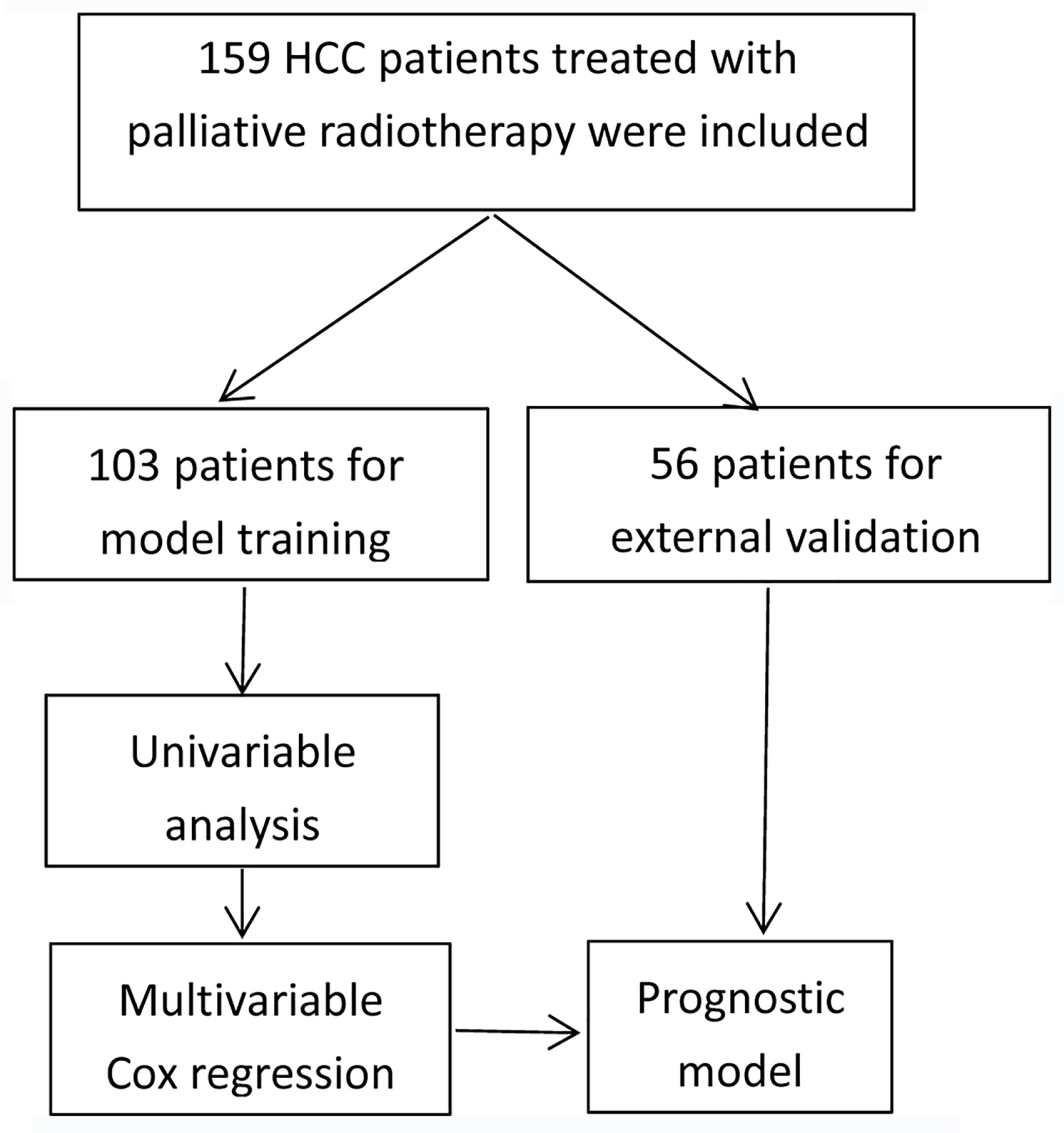
Flowchart of patients inclusion.

The detailed patients inclusion process was illustrated in **Figure 1**. Briefly, 159 patients who met the inclusion criteria were finally included, with a median age of 55 years (range, 24-84 years). Men accounted for 85.5% and women accounted for 14.5%. All patients were diagnosed by pathology. One hundred and three patients were included for predictive model training, while other 56 patients treated with palliative radiotherapy in the same time-period were used for external validation. The median follow-up time was 13.7 months (range: 0.7-64.2 months). The baseline demographic characteristics of the patients were shown in **Table 1**. Most patients have evident symptoms including pain, shortness of breath, abdominal discomfort, nausea, or fatigue. After palliative radiation treatment, about half of patients have improvement in symptoms. Most patients have no or grade I-II toxicity. Grade III blood toxicity was recorded in 2 patients and no patients have grade IV toxicity.

**Table 1:** Clinical characteristics of patients.

Clinical characteristics	Training set (n=103) N (%)	Validating set (n=56) N (%)
Sex (Male/Female)	88 (85.4%)/15 (14.6%)	48 (85.7%)/8 (14.3%)
Age at radiotherapy (< 55/≥ 55 years)	49 (47.6%)/54 (52.4%)	37 (66.1%)/19 (33.9%)
Tobacco (No/Yes)	78 (75.7%)/25 (24.3%)	37 (66.1%)/19 (33.9%)
Alcohol (No/Yes)	59 (57.3%)/44 (42.7%)	35 (62.5%)/21 (37.5%)
Viral hepatitis		
No	22 (21.4%)	15 (26.8%)
B type	76 (73.8%)	36 (64.3%)
Other types	5 (4.8%)	5 (8.9%)
Cirrhosis (No/Yes)	48 (46.6%)/55 (53.4%)	26 (46.4%)/30 (53.6%)
Chinese stage (IIIB/IV)	95 (92.2%)/8 (7.8%)	52 (92.9%)/4 (7.1%)
Tumor sites in liver		
Left lobe	17 (16.5%)	10 (17.9%)
Right lobe	52 (50.5%)	32 (57.1%)
Caudate lobe	11 (10.7%)	3 (5.4%)
≥ 2 lobes	23 (22.3%)	11 (19.6%)
Diagnostic type (Pathological/Clinical)	68 (66.0%)/35 (34.0%)	23 (41.1%)/33 (58.9%)
Metastatic sites		
Liver (No/Yes)	34 (33%)/69 (67%)	21 (37.5%)/35 (62.5%)
Bone (No/Yes)	55 (53.4%)/48 (46.6%)	34 (60.7%)/22 (39.3%)
Lung (No/Yes)	54 (52.4%)/49 (47.6%)	31 (55.4%)/25 (44.6%)
Others (No/Yes)	47 (45.6%)/56 (54.4%)	29 (51.8%)/27 (48.2%)
PVTT (No/Yes)	80 (77.7%)/23 (22.3%)	31 (55.4%)/25 (44.6%)
IVCT (No/Yes)	96 (93.2%)/7 (6.8%)	50 (89.3%)/6 (10.7%)
Tumor in liver (No/Yes)	23 (22.3%)/80 (77.7%)	10 (17.9%)/46 (82.1%)
AFP (Normal/High)	70 (68.0%)/33 (32.0%)	35 (62.5%)/21 (37.5%)
Early therapies		
Surgery (No/Yes)	47 (45.6%)/56 (54.4%)	36 (64.3%)/20 (35.7%)
Radiofrequency ablation (No/Yes)	79 (76.7%)/24 (23.3%)	46 (82.1%)/10 (17.9%)
Intervention therapy (No/Yes)	65 (63.1%)/38 (36.9%)	37 (66.1%)/19 (33.9%)
Target therapy (No/Yes)	87 (84.5%)/16 (15.5%)	51 (91.1%)/5 (8.9%)
Chemotherapy (No/Yes)	81 (78.6%)/22 (21.4%)	49 (87.5%)/7 (12.5%)
BED (< 60 Gy/≥ 60 Gy)	49 (47.6%)/54 (52.4%)	16 (28.6%)/40 (71.4%)
Radiation dose (< 40/≥ 40 Gy)	44 (42.7%)/59 (57.3%)	14 (25.0%)/42 (75.0%)
Fraction (Conventional/SBRT)	39 (37.9%)/64 (62.1%)	17 (30.4%)/39 (69.6%)
Radiation of tumor in		
Liver	28 (27.2%)	17 (30.4%)
Bone	28 (27.2%)	16 (28.6%)
Lung	26 (25.2%)	14 (25.0%)
Others	21 (20.4%)	9 (16.1%)
Target therapy combination (No/Yes)	97 (94.2%)/6 (5.8%)	53 (94.6%)/3 (5.4%)
Chemotherapy combination (No/Yes)	91 (88.3%)/12 (11.7%)	50 (89.3%)/6 (10.7%)
Toxicity		
Hepatic (No/Yes)	91 (88.3%)/12 (11.7%)	53 (94.6%)/3 (5.4%)
Gastroenterological (No/Yes)	89 (86.4%)/14 (13.6%)	53 (94.6%)/3 (5.4%)
Hematological (No/Yes)	82 (79.6%)/21 (20.4%)	52 (92.9%)/4 (7.1%)

PVTT, portal vein tumor thrombus; IVCT, inferior vena cava thrombosis; AFP, alpha-fetoprotein; BED, biologically effective dose; SBRT, stereotactic body radiation therapy.

### Univariable Cox Regression Analyses

**Figure 2:**
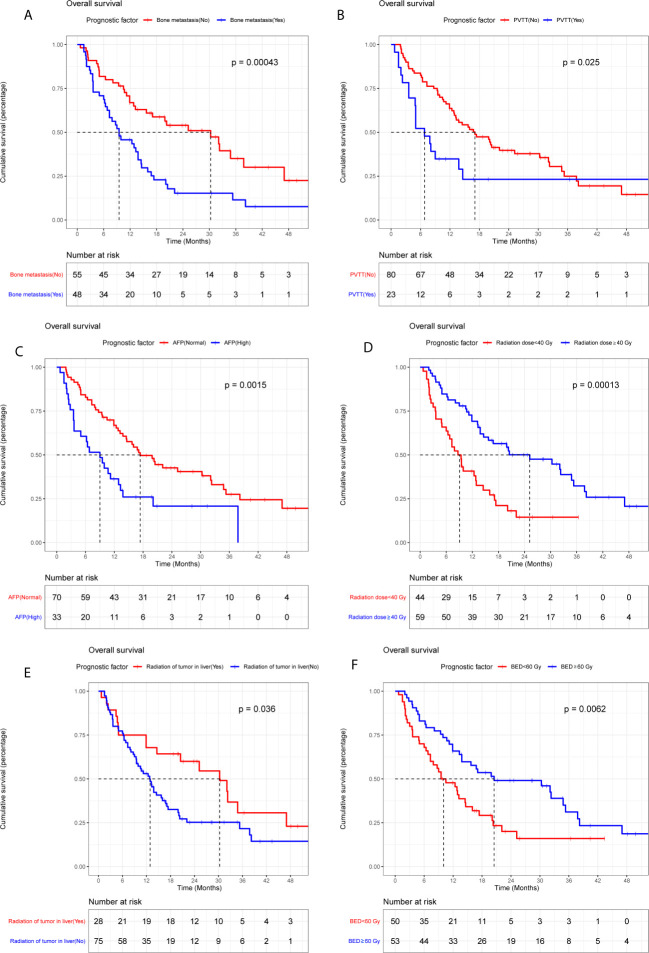
Kaplan-Meier plots of overall survival stratified by bone metastasis **(A)**, portal vein tumor thrombus **(B)**, alpha-fetoprotein **(C)**, radiation dose **(D)**, radiation of tumor in liver **(E)**, and BED **(F)**.

The median OS of LC patients receiving palliative radiotherapy is 14.8months (**Supplementary Figure 1**). In model training phase, thirty-one characteristics were investigated by Kaplan-Meier curves and log-rank tests. Six factors (bone metastasis, portal vein tumor thrombus, alpha-fetoprotein, radiation of tumor in liver, radiation dose and biologically effective dose) were considered potentially significant (*P* < 0.05) (**Table 2** and **Figure 2**). Patients with bone metastasis have a median OS of 9.5 months, which is shorter than patients without bone metastasis (17.1 months). Patients with portal vein tumor thrombus (PVTT) have a median OS of 6.8 months, while the median OS of patients without PVTT is 15.1 months. Patients with and without high alpha-fetoprotein (AFP) have a median OS of 9.0 and 16.4 months, respectively. Patients with low radiation dose (< 40 Gy) have a shorter median OS (6.6 months) than patients with high radiation dose (≥40 Gy) (16.8 months). Patients with radiation of tumor in liver have longer survival (30.2 months) compared to patients without radiation of tumor in liver (12.9 months). Patients with higher biologically effective dose (BED ≥ 60 Gy) have a better median OS (20.6 months) compared to patients with low BED (< 60 Gy) (10.1 months).

**Table 2:** Univariable and multivariable Cox regression analysis.

Characteristics	Univariable (*P* value)	Multivariable Cox regression analysis		
		HR	95%CI	*P *value
Bone metastasis (Yes)	0.000	1.781	1.070-2.966	0.026
PVTT (Yes)	0.025	2.078	1.150-3.755	0.015
AFP (High)	0.002	2.098	1.220-3.608	0.007
Radiation of tumor in liver (Yes)	0.036	1.168	0.605-2.257	0.644
Radiation dose ≥ 40 Gy	0.000	0.535	0.311 -0.919	0.023
BED ≥ 60 Gy	0.006	–	–	–

PVTT, portal vein tumor thrombus; AFP, alpha-fetoprotein; BED, biologically effective dose.

### Multivariable Cox Regression Analyses

BED was excluded in multivariable Cox regression analysis because of the high co-linearity between BED and radiation dose (*P*< 0.05). Thereafter, the remaining five variables were included in the multivariable Cox regression model. Four factors including bone metastasis (HR = 1.781, *P* = 0.026), PVTT (HR = 2.078, *P* = 0.015), AFP (HR = 2.098, *P* = 0.007) and radiation dose (HR = 0.535, *P* = 0.023) have significant potential to predict survival (**Table 2**). Harrell’s C-index of the predictive model is 0.709 (95%CI, 0.649-0.769), which indicates good discriminative ability. Additionally, calibration curves of the 1- and 2-year OS of the predictive model indicate that the predicted probabilities of OS are very close to the actual observed outcomes (**Supplementary Figure 2**). For external validation, the Harrell’s C-index is 0.735 (95%CI, 0.666-0.804) and calibration curves also indicate good feasibility of the predictive model (**Supplementary Figure 3**).

### Assessment of Robustness of Predictive Model by Stratified Analyses

Two radiation treatment techniques including conventional fraction and stereotactic body radiation therapy (SBRT) were used for included patients during the study period. The OS of these two groups of patients have no statistical difference (**Supplementary Figure 4**). Stratified analyses show that predictive model based on bone metastasis, PVTT, AFP, and radiation dose was robust both for patients treated with SBRT or conventional fraction radiation (**Supplementary Table 1**). Metastatic tumors in different organs including liver, lung, bone, and others were radiated. To further investigate the robustness of our predictive model for patients with metastatic tumors in different organs, stratified analyses were conducted and the results demonstrate that our predictive model perform good for all patients (**Supplementary Table 1**).

### Nomograms for Survival of LC Patients

**Figure 3:**
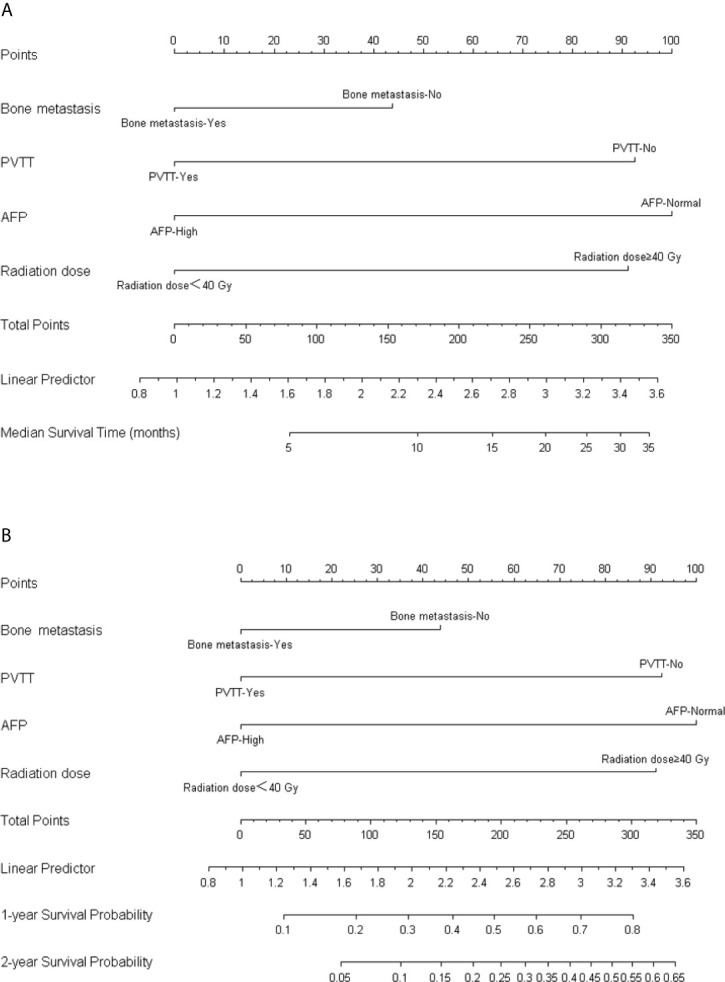
Nomogram for predicting the median OS **(A)** and 1-year/2-year survival probability **(B)**.

Coefficients obtained from the multivariable Cox regression model were taken to establish nomograms for median survival time and 1- and 2-year OS probability (**Figure 3**). Each variable included in the model was assigned a score by locating it to the point scale. The total score of all the variables determines the prediction of a patient’s outcome by drawing a vertical line from the total score to the median survival time scale and survival probability scale, respectively. As shown in the **Figure 3**, more total score means better prognosis of patients.

## Discussion

To prevent LC patients receiving palliative radiation from over- or under-treatment, survival prediction of patients should be considered before decision making. In this study, 6 clinical characteristics were indicated to be associated with OS of patients according to univariable analysis, and 4 (bone metastasis, PVTT, AFP and radiation dose) of them were statistically significant in multivariable Cox regression analysis.

Bone metastasis often leads to skeletal-related events, such as severe bone pain, pathological fractures, spinal cord compression and hypercalcemia. Habermehl et al.’s study showed that the median OS of LC patients with bone metastasis was 4.2 months after palliative radiation (range, 0.2-38.9 months) (6). In line with Habermehl et al.’s study, we found that bone metastasis can significantly reduce the OS of LC patients (HR = 1.781, *P* = 0.026). Moreover, about 10%-40% of LC patients have macrovascular invasion (MVI) including portal and/or hepatic veins when they were initially diagnosed (12–14). MVI is an independent predictive factor of poor OS in LC patients. The median OS of LC patients with MVI is significantly lower than those without MVI (2-4 months vs.10-24 months). Similarly, in our study, PVTT can significantly reduce survival of LC patients (HR = 2.078, *P* = 0.015). Patients with PVTT have a median OS of 6.8 months, which is shorter than patients without PVTT (15.1 months). Furthermore, Elevated AFP before palliative radiotherapy is associated with poor survival. Numerous studies use AFP as a biomarker to predict survival of LC patients. Czauderna et al.’s study shows that high pre-treatment AFP predicts reduced OS in LC (15). In our study, high AFP before palliative radiotherapy also can be an indicator of poor OS (HR = 1.098, *P* = 0.007). In addition, our study shows that high radiation dose can reduce death rate of advanced liver cancer patients (HR = 0.535, *P* = 0.023). Similarly, in Kong et al.’s study, the median OS of LC patients treated with high-dose radiation was better than that patients with low-dose radiotherapy (42 months vs. 19 months) (16).

There are several limitations should be stated in our article. Firstly, our findings should be interpreted with caution due to the retrospective design of our study. Moreover, all the patients included in our study were treated in a single hospital, which means that potential selection bias may diminish the accuracy of our conclusions. Secondly, different early therapies including surgery, chemotherapy, Intervention therapy and targeted therapy may increase the heterogeneity of included patients. Thirdly, different radiation techniques including SBRT and conventional radiation were taken to treat patients. Although our stratified analyses indicate that our predictive model perform good both for patients treated with conventional radiation and SBRT, our model may fail to predict the OS of patients treated with other radiation techniques. Fourthly, elaborative data of symptoms and QoL was absent, leading to absence of quantitative assessment of symptoms and QOL improvement by scale tools. Finally, several patients received both of palliative radiation and target therapy/chemotherapy during the study time period. According to our univariable analysis, target therapy or chemotherapy combined with palliative radiation have no significant effect on the OS of LC patients compared with patients received single palliative radiation. However, this conclusion should be further investigated in the future because of the small number of patients (6 patients with target therapy and 12 patients with chemotherapy).

Conclusively, four predictive factors of survival of advanced LC patients treated with palliative radiotherapy were identified. These factors were bone metastasis, PVTT, AFP and radiation dose. Recommendations for an individualized palliative radiotherapy for advanced LC patients could be made based on these four factors.

## Data Availability Statement

The raw data supporting the conclusions of this article will be made available by the authors, without undue reservation.

## Author Contributions

QH, DZ, and YL analyzed the data and draft the manuscript. YH, PL, and GX contributed to the collection of data. TZ and JX contributed to the design of the research. All authors contributed to the article and approved the submitted version.

## Funding

JX is supported by research clinician grant of Tongji medical college, Huazhong university of science and technology (No. 5001530078). This work is supported by the National Natural Science Foundation of China (81903103 to DZ and 81874061 to TZ).

## Conflict of Interest

The authors declare that the research was conducted in the absence of any commercial or financial relationships that could be construed as a potential conflict of interest.

## Publisher’s Note

All claims expressed in this article are solely those of the authors and do not necessarily represent those of their affiliated organizations, or those of the publisher, the editors and the reviewers. Any product that may be evaluated in this article, or claim that may be made by its manufacturer, is not guaranteed or endorsed by the publisher.

## Supplementary Material

Supplementary Figure 1 Kaplan-Meier curve of overall survival of included patients.

Supplementary Figure 2 Calibration curves of the 1- **(A)** and 2-year **(B)** OS of the predictive model in training phase.

Supplementary Figure 3 Calibration curves of the 1- **(A)** and 2-year **(B)** OS of the predictive model in validation phase.

Supplementary Figure 4 Kaplan-Meier curves of overall survival of patients treated with SBRT or conventional radiation.
