# Supplementary figures and images for: Prognostic Factors of Survival of Advanced Liver Cancer Patients Treated With Palliative Radiotherapy: A Retrospective Study
Source: Front Oncol. 2021 Jul 28;11:658152. doi: 10.3389/fonc.2021.658152 (PMC8355619; doi:10.3389/fonc.2021.658152)

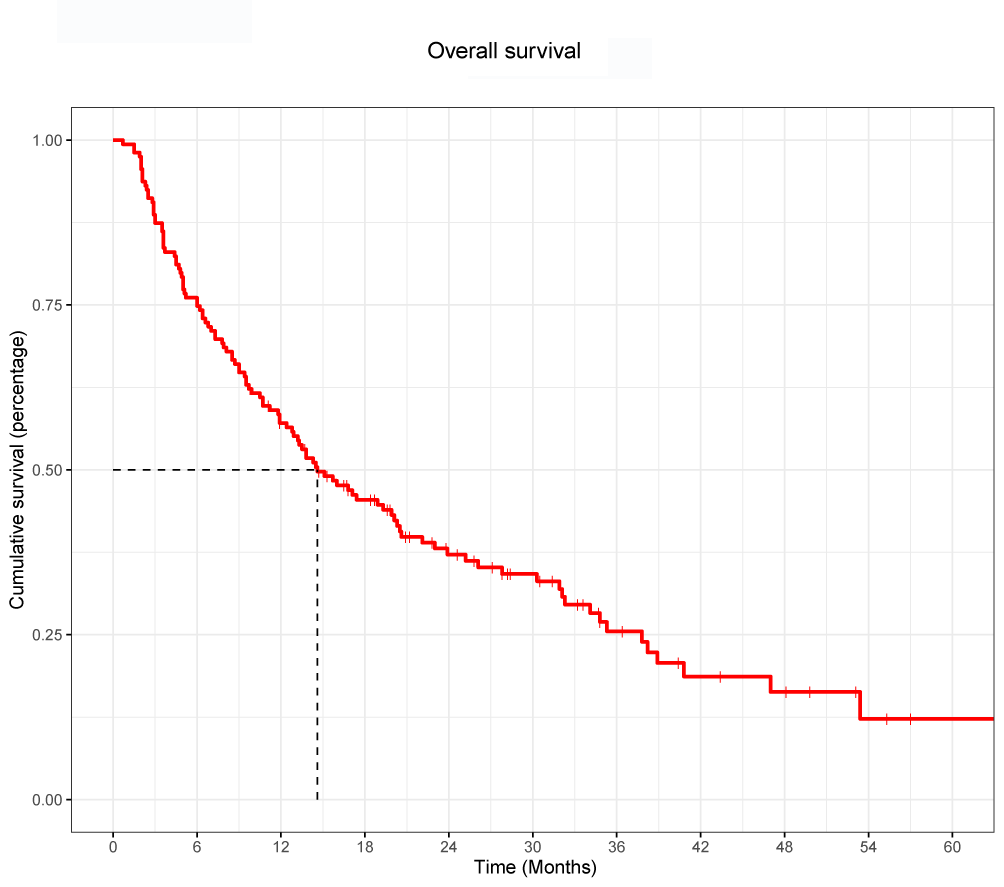

Supplement: Supplementary Figure 1 — Kaplan-Meier curve of overall survival of included patients. [file Image_1.tif]

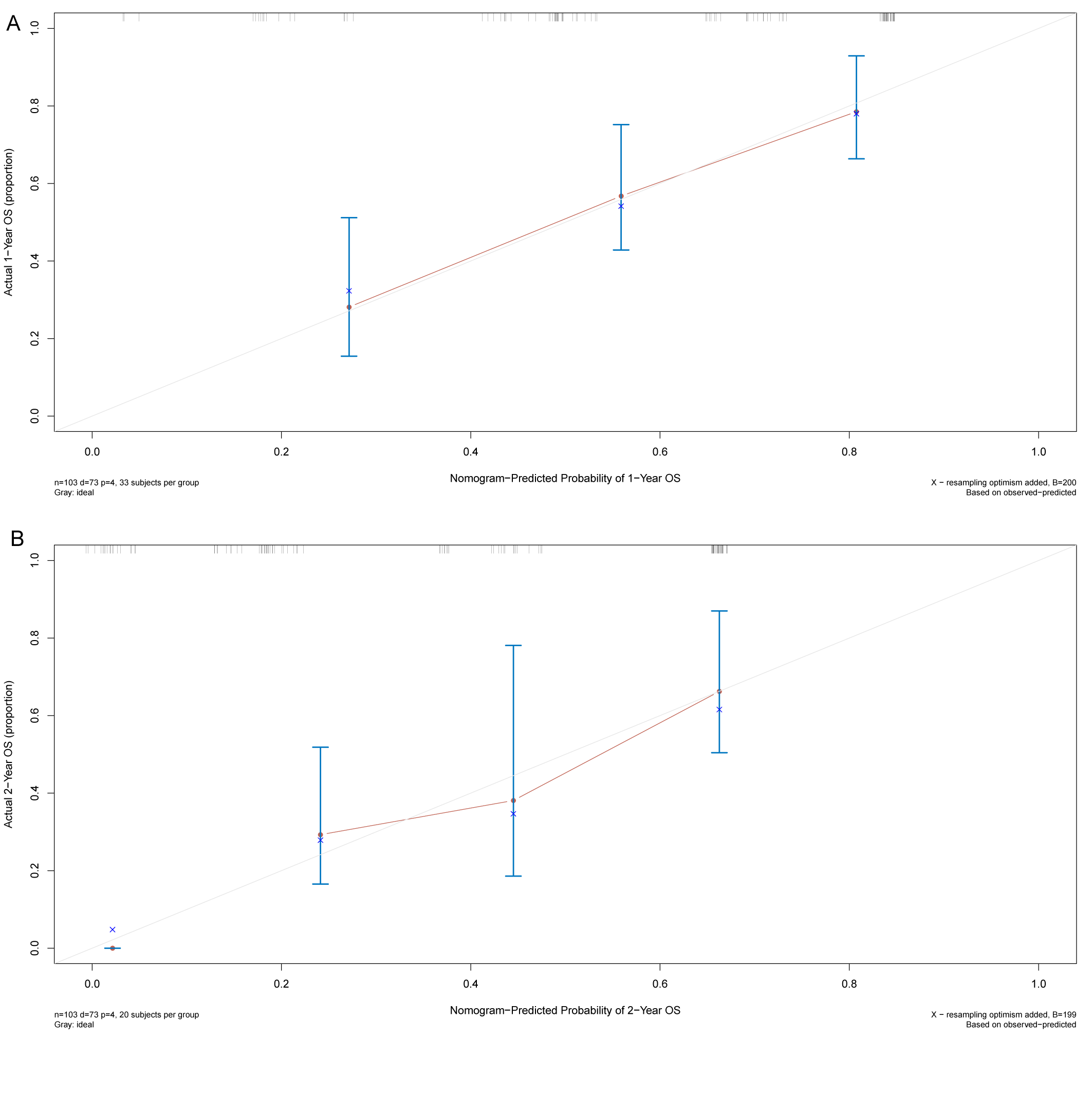

Supplement: Supplementary Figure 2 — Calibration curves of the 1- (A) and 2-year (B) OS of the predictive model in training phase. [file Image_2.tif]

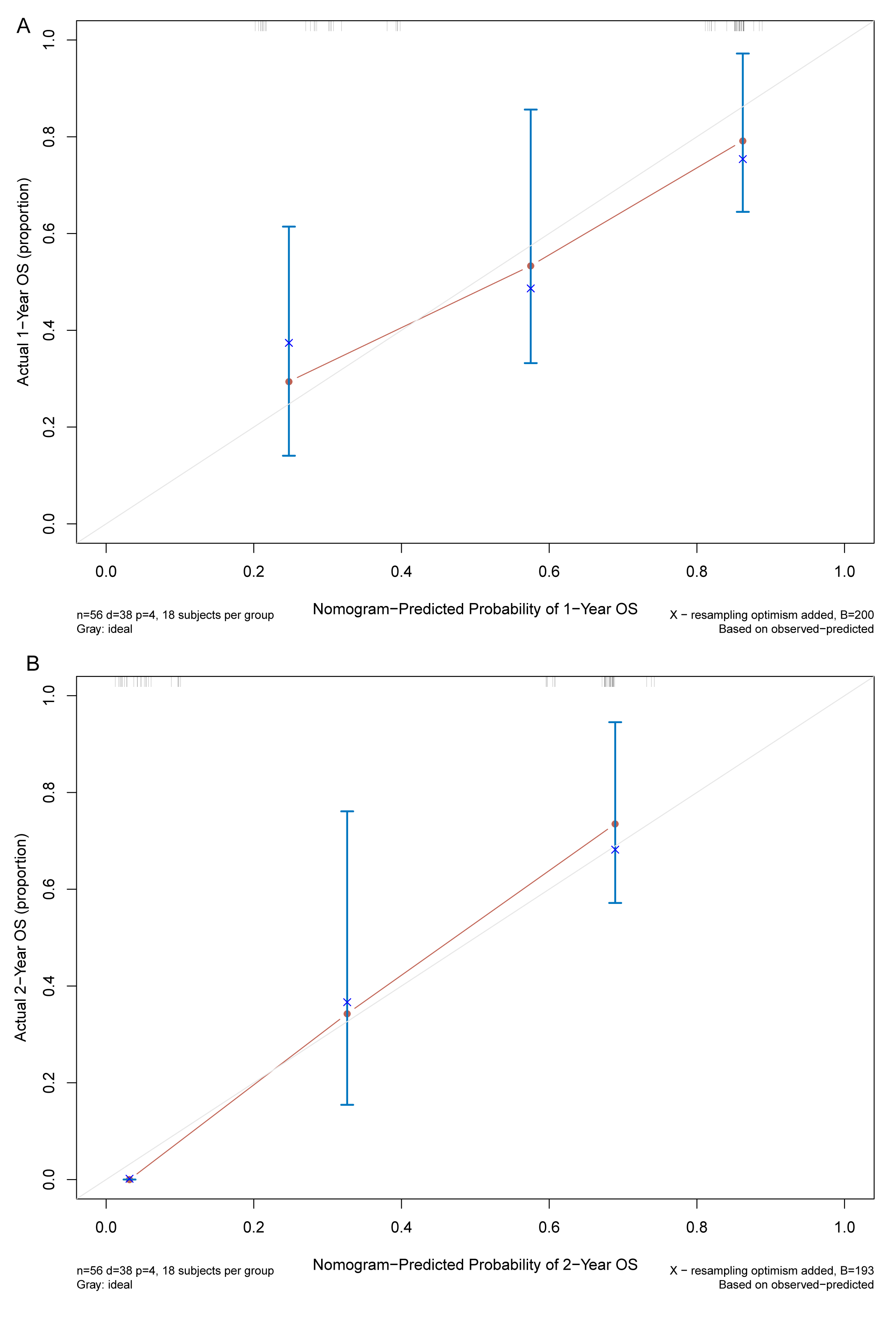

Supplement: Supplementary Figure 3 — Calibration curves of the 1- (A) and 2-year (B) OS of the predictive model in validation phase. [file Image_3.tif]

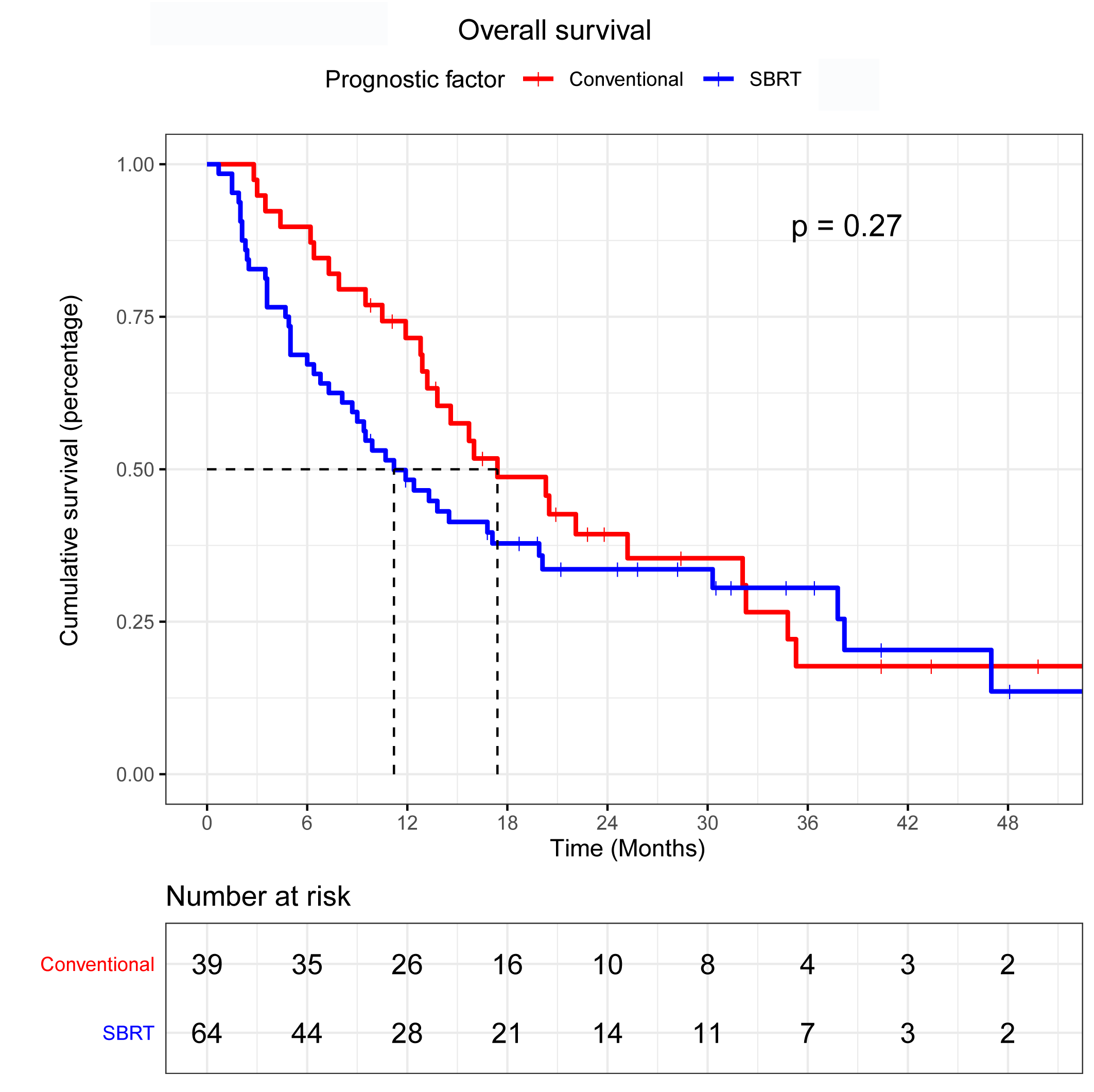

Supplement: Supplementary Figure 4 — Kaplan-Meier curves of overall survival of patients treated with SBRT or conventional radiation. [file Image_4.tif]
