# Supplementary material for: Prognostic Factors of Survival of Advanced Liver Cancer Patients Treated With Palliative Radiotherapy: A Retrospective Study
Source: Front Oncol. 2021 Jul 28;11:658152. doi: 10.3389/fonc.2021.658152 (PMC8355619; doi:10.3389/fonc.2021.658152)
Supplement: Supplementary file 5 [file Table_1.docx]

**Supplementary Table S1.** Assessment of predictive model by stratified analyses

| **Patient characteristics** | **HR (95%CI) *P* value** | | | | **Harrell's C-index(95%CI)** |
| --- | --- | --- | --- | --- | --- |
|  | **Bone metastasis** | **PVTT** | **AFP** | **Radiation dose** |  |
| **Fraction** |  |  |  |  |  |
| Conventional | 1.482 (1.189-2.052) 0.042 | 2.206 (1.537-2.961) 0.035 | 2.115 (1.336-2.658) 0.027 | 0.631 (0.396-0.814) 0.017 | 0.712(0.642-0.782) |
| SBRT | 1.674 (1.398-2.856) 0.015 | 2.471(1.993-2.854) 0.016 | 2.074 (1.266-2.893) 0.018 | 0.552 (0.207-0.768) 0.008 | 0.726(0.614-0.838) |
| **Radiation of tumor in** |  |  |  |  |  |
| Liver | 1.582(1.206-1.993) 0.027 | 2.915 (2.095-4.032) 0.017 | 2.106 (1.192-2.648) 0.039 | 0.495 (0.221-0.794) 0.015 | 0.706(0.615-0.797) |
| Bone | 1.424(1.380-2.425) 0.006 | 1.977 (1.383-2.982) 0.004 | 1.894 (1.347-2.365) 0.006 | 0.583 (0.395-0.831) 0.026 | 0.813(0.751-0.875) |
| Lung | 1.662(1.238-2.064) 0.029 | 2.013 (1.576-2.994) 0.019 | 2.231 (1.134-3.015) 0.042 | 0.412 (0.292-0.695) 0.004 | 0.721(0.597-0.845) |
| Others | 1.487(1.192-2.245) 0.038 | 2.096 (1.365-3.024) 0.025 | 1.784 (1.267-2.513) 0.036 | 0.675 (0.490-0.912) 0.048 | 0.705(0.623-0.787) |

PVTT, portal vein tumor thrombus; AFP, alpha-fetoprotein; SBRT, stereotactic body radiation therapy
